# The @RISK Study: Risk communication for patients with type 2 diabetes: design of a randomised controlled trial

**DOI:** 10.1186/1471-2458-10-457

**Published:** 2010-08-05

**Authors:** Laura MC Welschen, Sandra DM Bot, Jacqueline M Dekker, Daniëlle RM Timmermans, Trudy van der Weijden, Giel Nijpels

**Affiliations:** 1 Department of General Practice, EMGO Institute for Health and Care Research, VU University Medical Center, Amsterdam, the Netherlands; 2 Department of Epidemiology and Biostatistics, EMGO Institute for Health and Care Research, VU University Medical Center, Amsterdam, the Netherlands; 3 Department of Public and Occupational Health, EMGO Institute for Health and Care Research, VU University Medical Center, Amsterdam, the Netherlands; 4 Department of General Practice, CAPHRI School for Public Health and Primary Care, Maastricht University, Maastricht, the Netherlands

## Abstract

**Background:**

Patients with type 2 diabetes mellitus (T2DM) have an increased risk to develop severe diabetes related complications, especially cardiovascular disease (CVD). The risk to develop CVD can be estimated by means of risk formulas. However, patients have difficulties to understand the outcomes of these formulas. As a result, they may not recognize the importance of changing lifestyle and taking medication in time. Therefore, it is important to develop risk communication methods, that will improve the patients' understanding of risks associated with having diabetes, which enables them to make informed choices about their diabetes care.

The aim of this study is to investigate the effects of an intervention focussed on the communication of the absolute 10-year risk to develop CVD on risk perception, attitude and intention to change lifestyle behaviour in patients with T2DM. The conceptual framework of the intervention is based on the Theory of Planned Behaviour and the Self-regulation Theory.

**Methods:**

A randomised controlled trial will be performed in the Diabetes Care System West-Friesland (DCS), a managed care system. Newly referred T2DM patients of the DCS, younger than 75 years will be eligible for the study. The intervention group will be exposed to risk communication on CVD, on top of standard managed care of the DCS. This intervention consists of a simple explanation on the causes and consequences of CVD, and possibilities for prevention. The probabilities of CVD in 10 year will be explained in natural frequencies and visualised by a population diagram. The control group will receive standard managed care. The primary outcome is appropriateness of risk perception. Secondary outcomes are attitude and intention to change lifestyle behaviour and illness perception. Differences between baseline and follow-up (2 and 12 weeks) between groups will be analysed according to the intention-to-treat principle. The study was powered on 120 patients in each group.

**Discussion:**

This innovative risk communication method based on two behavioural theories might improve patient's appropriateness of risk perception and attitude concerning lifestyle change. With a better understanding of their CVD risk, patients will be able to make informed choices concerning diabetes care.

**Trail registration:**

The trial is registered as NTR1556 in the Dutch Trial Register.

## Background

Patients with type 2 diabetes mellitus (T2DM) have an increased risk to develop severe complications including cardiovascular disease (CVD). The risk of these complications can be reduced by an adequate treatment with medication and by adopting a healthy lifestyle [1-3]. During the last few decades, patients are encouraged to become more actively involved in diabetes care. It is now believed that they should be the primary decision-makers in the control of their diabetes as laid down in the patient empowerment approach [4,5]. According to this approach, patients should be encouraged to use their own ability to gain mastery over their diabetes.

Informing patients on the risks to develop severe diabetes related complications enables them to make informed choices. It has been shown that patients underestimate these risks, and that they do not understand the explanation of caregivers about risks [6-13]. In contrast, it was also found that some patients are unrealistically pessimistic about their risk to develop CVD, resulting in anxiety [14,15]. Both optimistic and pessimistic patients have a reluctance in self-management [12-14,16,17]. The risk of developing complications should be explained in a way that is understandably for patients, to give them opportunities to make informed choices on their diabetes care. With clear information on the risk of developing complications, patients' risk perception may improve, which may result in a change in their attitude and intention to change lifestyle behaviour [12,14,17,18]. Although some caregivers believe that patients do not desire to take responsibility for their disease and that this responsibility might harm them [19], there is evidence that patients welcome the given responsibility. It is still unclear though how this can be achieved [20].

### Risk communication

Healthcare professionals usually explain numerical risks of developing diabetes-related complications, in percentages, frequencies, relative risks, absolute risk and 'number needed to treat' [7,21]. However, patients often do not understand the meaning of these numbers [10,11].

Specific insights on how to communicate health risks to patients have become available. Firstly, it is important to provide a clear and very simple message. This message should consist of information on what causes the risk, what are the consequences of the risk, and what can be done to prevent or treat the problem [22-26]. Secondly, the message should consist the individual risk probability in formats that patients are more likely to understand such as a visual presentation of risks rather than a presentation in percentages [18,21,23,27-32]. It is not clear what timeline for the risk presentation is preferred, but most absolute risks on CVD are predicted in a 10-year time horizon. Thirdly, positive framing, which means that the benefits of behaviour change are highlighted instead of a frame that focuses on the effect of not changing in terms of loss of healthy years of one's life, seems to help to increase patients' motivation [23,28,32-34].

In this study, we will use this broad approach of risk communication; we will not only focus on the communication of the actual risk but also on the causes and consequences of the risk by using the principles of a simple but complete message, visual representation and a positive framework to investigate if patients are able to understand the meaning of risks.

### Theoretical framework

The importance of risk communication for patients with T2DM can be explained by means of a theoretical framework. The idea is that patients are not willing to change their lifestyle if they are not informed on the reason why they should change. It is believed that people have perceptions concerning their disease, either correct or incorrect. These perceptions determine how people cope with their disease and how they manage their risks to develop severe complications. The hypothesis is that by providing understandable information on the disease by means of risk communication illness perceptions will change and, in addition, the attitude of patients concerning the importance of behaviour change.

The conceptual framework for this idea is based on two theories: the Self-regulation theory of Leventhal [35-37] and the Theory of Planned Behaviour [38,39].

Leventhal's Self-regulation Theory assumes that a patient's perceptions on the impact of diabetes and its treatment determine the attitude concerning self-management. Illness perceptions include the identity, timeline, cause, consequences and controllability of the disease [35-37,40].

The attitude to change behaviour is described in the second theory, the Theory of Planned Behaviour [38,39]. According to this theory, there are three determinants of intention to change a specific behaviour: a) attitude towards the behaviour; b) subjective norm which represent perceived social pressure by significant others to perform the behaviour, and c) perceived behavioural control, which refers to the perceived ease or difficulty to perform the behaviour. For the framework of this study, it is expected that risk communication will have impact on the attitude to change behaviour.

The plausibility of our theoretical framework will be established by measuring the components of the theories by means of questionnaires.

### Objectives

The primary objective of this study is to investigate the effect of a risk communication intervention on appropriateness of risk perception in patients with T2DM. Secondary objectives are to investigate the effects on illness perceptions, attitude, and intention to change behaviour. We will also assess patients' general satisfaction with the communication and anxiety to check for adverse effects of risk communication.

## Methods

### Design of the study

The study is a randomised controlled trial. The Medical Ethical Committee of the VU University Medical Center in Amsterdam approved the study protocol.

### Setting

The trial is conducted within 'the Diabetes Care System (DCS) West-Friesland', a managed care system that was implemented in The Netherlands in 1997. The DCS was described previously [41]. Briefly, this care system provides additional care to that of the general practitioners (GPs) in the region West-Friesland of the Netherlands. GPs in the region refer all their T2DM patients to the DCS. Each patient visits the DCS annually for a physical examination, followed by a visit to a diabetes nurse and a dietician for information and advice on their diabetes treatment. The results of this annual examination are sent to the patient's GP, who is responsible for the management of the patient and delegation of tasks to their practice nurse, according to the guidelines of the Dutch College of General Practitioners [42]. These guidelines recommend every patient with diabetes to visit a GP every 3 months. The well-organised infrastructure of the DCS with experienced medical assistants, diabetes nurses, and dieticians provides a suitable setting to implement a new intervention for patients with diabetes.

### Study population

The study population consist of newly referred patients with type 2 diabetes to the DCS. Inclusion criteria are: maximum age of 75 years and capable to fill in questionnaires in the Dutch language. Patients that have experienced a cerebrovascular accident or transient ischaemic attack will be excluded from participation in the study because they might have communication problems. Patients with other CVD may participate in the study.

Patients that fulfil the inclusion criteria will be sent an information letter as well as a concept of the informed consent form of the study. Subsequently, the patient will visit the medical assistant for their annual physical examination of the DCS concerning their standard care. In this visit, the medical assistant will also explain the purpose of the study and interest to participate in the study will be asked. All participating patients must sign a written informed consent form. After that, physical measurements are performed, which include body weight, blood pressure, and drawing of blood samples (to assess fasting blood glucose, HbA1c, total cholesterol, HDL-cholesterol and triglycerides). In addition, patients are given the first self-administrated questionnaire to fill in at the DCS.

### Treatment allocation

**Figure 1:**
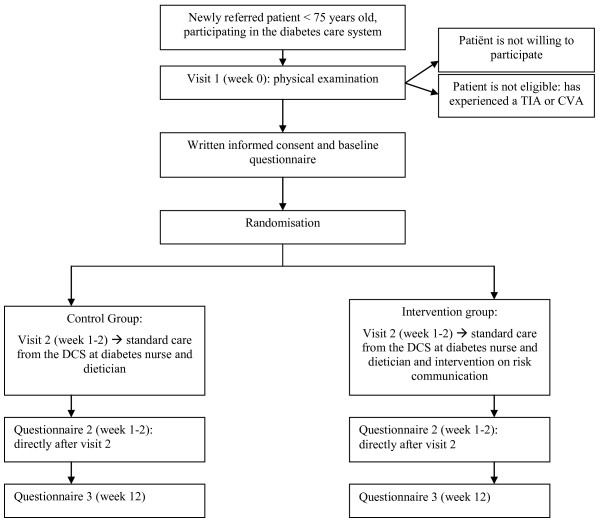
**Design of the randomised controlled trial on the effects of risk communication in patients with type 2 diabetes**.

For intervention allocation a randomization list is drawn up using a computerized randomization computer program (Random Allocation Software version 1.0.0). Patients are randomly assigned to either the intervention group, receiving the risk communication intervention in addition to the managed diabetes care or the control group, receiving managed diabetes care only. Approximately two weeks after the first visit, patients are scheduled for either the intervention or control session to the diabetes nurse and dietician. The manager of the DCS, who is not involved in the patients' care, allocates the patient to one of the two groups on the basis of the randomisation list. The flow of the patients will be registered by a medical assistant according to a flow diagram recommended by the CONSORT statement [43]. Participants may withdraw from the study at their own request and without providing reasons. Provided reasons for withdrawal will be registered. Figure 1 shows the design of the study.

### Blinding

Patients, diabetes nurses and dieticians cannot be blinded to the intervention. The principal investigator (LMCW) remains blinded during the entire intervention.

### Interventions

#### Control group

Patients allocated to the control group receive managed diabetes care provided by the DCS. Patients are invited for a physical examination at the DCS. One or two weeks later, the patient visits a diabetes nurse and dietician, for 30 minutes each, in order to receive results from the physical examination, including an explanation of high and low values of the different lab results such as HbA1c and cholesterol, and general information and education on diabetes problems, dietary intake and physical activity.

Follow-up visits to the DCS are optional if desired. In addition, the patients will visit the GP every 3 months according to the guidelines of the Dutch College of General Practitioners [42].

#### Intervention group

Patients who are assigned to the intervention group will receive a risk communication intervention in addition to the managed care of the DCS. This means that these patients will also be invited for a physical examination, followed by a visit to the diabetes nurse and dietician, also for 30 minutes each. This visit will consist of the risk communication intervention (see below) followed by standard care of DCS. The diabetes nurses and dieticians will receive training in performing the intervention. Follow-up visits to the DCS are optional for patients in the intervention group as well. The risk communication intervention will not be performed again in follow-up visits. In addition, these patients will also visit the GP every 3 months according to the guidelines of the Dutch College of General Practitioners [42].

The risk communication consists of the previously described principles: a simple message including the cause and consequences of the risk and possibilities to improve the risk, explaining the absolute 10-year risk in natural frequencies and visual representation (population diagram), and positive framing (gain versus loss).

The intervention starts at the diabetes nurse. By means of nine variables (age at diagnosis, duration of diabetes, sex, ethnicity, smoking status, systolic blood pressure, HbA1c, total cholesterol, and HDL-cholesterol) the risk to develop CVD will be estimated with the UKPDS risk engine [44]. The diabetes nurse will explain the meaning of this risk according to an intervention protocol with six steps:

##### 1) The introduction of the risk communication

This step includes a general explanation about health related risks concerned with T2DM, including the cause and consequences of the CVD risk. This step is focused on the dimension 'cause' and 'consequences' from the Self-regulation Theory [35].

##### 2) Communication of the absolute risk according to the UKPDS risk engine

In this step, the absolute risk to develop CVD in the next 10 years will be explained in the format of natural frequencies. For example, the following sentence will be used: "Your risk to develop CVD in the next 10 years is X%. This means that of the 100 men/women of your age and with the same lab results and who also smoke/do not smoke, × will develop CVD in the next 10 years".

##### 3) Visual communication by means of a risk card

In this step, the risk will be explained again with the help of a population diagram. This diagram shows 100 people and the diabetes nurse indicates which people will develop CVD in the next 10 years.

##### 4) Positive framing: explanation that lifestyle changes can help to reduce the risk

The following step is 'message framing': translate the risk estimation into a positive message. The risk has a negative character but in this step will be explained that the patient is able to change the risk. In addition, the message will include the possibilities of how to change the risk. It is estimated that the risk to develop CVD in the next 10 years can be reduced by 30% by changing lifestyle habits, such as increasing physical activity, eating a healthier diet and to quit smoking. For example, a risk of 30% can be reduced to 21%. This means that the absolute health gain is 9%. The diabetes nurses will show this on the risk card by indicating the people that will no longer be at risk to develop CVD. In this example, 9 people will be indicated. This step is based on the dimension 'controllability' of the Self-regulation Theory [35].

##### 5) Communication with the patient for a reaction

After having finished the explanation of the risk, the diabetes nurse will ask the patient to give a reaction on the information that has been given using open questions.

##### 6) Think aloud: patient has to explain the risk him/herself

The patient will be encouraged to 'think aloud' about the risk and the meaning of the risk. It is believed that active participation will enable the patient to remember the information more easily [45].

Subsequently, the patient visits the dietician, who will continue the intervention. The dietician will start with step 6 of the intervention, the 'think aloud method' to see if the patient is able to explain what he/she has learned about his/her CVD risk. Depending on the answer of the patient, the dietician will choose one of the next steps:

• If the patient explains the CVD risk and what he/she can do to change the risk accurately, the dietician will confirm this. The remaining time of the visit will then be used for general diabetes related information and education on dietary intake and physical activity.

• If the patient is not able to explain the CVD risk or the possibilities of changing the risk, the dietician will repeat the risk communication, starting at step 2. Next, the dietician will provide usual care of the DCS on dietary intake and physical activity.

All steps will carefully be registered in order to use this information in the analysis.

Training of the diabetes nurses and dieticians

The diabetes nurses and dieticians received a training consisting of the following subsequent parts:

1) Training 1: a half day training by an experienced communication coach in health care settings and the principal investigator (LMCW). The training consisted of an introduction on the importance of providing information to the patients on risks and theory on risks communication, including the definition of a risk, an explanation of absolute and relative risks, methods of risk communication and the Self-regulation Theory and Theory of Planned Behaviour. In addition, the UKPDS risk engine was explained including the scientific background of its development. The last part of this day consisted of an explanation of the intervention. In order to increase treatment fidelity to the intervention, attention was also paid to the usual care for the control group, which is standardised by means of a protocol. This training day ended with practising the intervention steps by means of role plays.

2) Pilot study: a pilot study was performed to give diabetes nurses and dieticians the opportunity to learn their skills. The coach attended visits with two patients of each diabetes nurse and dietician ('coaching-on-the-job'). She checked all steps of the intervention and the general quality of the communication and provided feedback immediately after each visit.

3) Training 2: another half day by the coach and principal investigator. During this training the coach discussed the most important issues that occurred during the pilot study to the whole group.

The training was guided by a handbook developed by the researchers and the coach including the theoretical background of risks and risk communication, the protocol for the intervention and control group, the risk card, and the role plays.

### Treatment fidelity

All diabetes nurses and dieticians were trained to perform the intervention and they all have contact with patients of both the intervention and control group. Due to practical reasons, (i.e the part-time availability of the diabetes nurses and dieticians) it was not possible to divide the caregivers into two groups.

To increase treatment fidelity we will take two measures. Firstly, we developed a protocol for both the intervention and control group that is obliged to use during the whole study period by all diabetes nurses and dieticians. Secondly, 20 tape-recordings (10 intervention patients and 10 control patients) are made to assess treatment fidelity of the diabetes nurses and dieticians.

### Outcome assessment

Primary and secondary outcome measurements are assessed at baseline, and again after 2 weeks (directly after the intervention or control visit to the diabetes nurse and dietician) and 12 weeks by means of self-reported questionnaires.

#### Primary outcome measure

Appropriateness of risk perception is the primary outcome measure. Risk perception will be measured by using a question from a questionnaire previously developed for the IMPALA study [46]. This question is 'If the mean risk of developing CVD in the next 10 years for men with diabetes is 20 of 100 men and for women 15 of 100 women, how would you rate your risk of developing CVD in the next 10 years?' In addition, at 2 and 12 weeks only, a population diagram will also be shown in the questionnaire. Patients will be asked to indicate how many of 100 people will develop CVD in the next 10 years.

Appropriateness of risk perception will be assessed by a comparison of the UKPDS score [44] of the patient with the risk perception. Appropriateness is considered as a patient with a high-risk perception while indeed having a high risk, or a patient with a low-risk perception while having a low risk [46]. According to the UKPDS risk engine, a risk of > 30% is considered high risk, a risk between 15 and 30% is an intermediate risk, a risk < 15% is a low risk.

#### Secondary outcome measures

1. Anxiety and worry about CVD risk is assessed by a 7-point Likert scale, ranging from 'not anxious/worried at all' to 'very worried/anxious'. These questions were used in an earlier study by Claassen et al. [13].

2. Illness perception is assessed by the Brief Illness Perception Questionnaire (Brief-IPQ) [47]. The Brief-IPQ consist of 8 items on the seriousness and impact of diabetes on various aspects of life, measured on a 10-point Likert scale ranging from 'strongly disagree' to 'strongly agree'. There is also 1 open question that asks patients to list what they consider the three most important causes for their disease.

3. Attitude and intention to change behaviour according to the Theory of Planned Behaviour [38] are measured by the Determinants of Lifestyle Behaviour Questionnaire, which was developed for a randomised controlled trial to investigate the effects of a cognitive behaviour programme on the primary prevention of T2DM and CVD [48]. The questionnaire consists of three parts, namely on the attitude and intention to change 1) dietary intake, 2) physical activity and 3) smoking behaviour. Each part has seven attitude items and three items on intention to change the specific behaviour. The attitude items have a 7-point scale. Examples of attitude items are: 'I consider eating healthier/increasing physical activity/quitting smoking good-bad, difficult-easy, frustrating-satisfactory'. The intention to change behaviour items have a 5-point Likert scale ranging from 'strongly agree' to 'strongly disagree'. An example is 'I intend to eat healthier/increase physical activity/stop smoking within two months'.

4. The Short Form Spielberger State Anxiety Inventory (SF-STAI) [49] consists of six items to assess the extent to which patients feel 'calm', 'tense', 'upset', 'relaxed', 'content', and 'worried' on a 4-point scale ranging from 'not at all' to 'very much'. Sum scores will range between 20 and 80 with higher scores indicating higher levels of anxiety.

5. Satisfaction with the communication is assessed by questions from the COMRADE scale [46]. This scale consists of 10 questions on a 5-point scale ranging from 'strongly disagree' to 'strongly agree'. These questions are only included in the second questionnaire, which is given at 2 weeks just after the visit to the diabetes nurse and dietician.

### Additional measurements

Physical examinations will be performed and blood samples are taken at baseline by medical assistants of the DCS at baseline in order to estimate the risk to develop cardiovascular disease by means of the UKPDS risk engine [44]. Systolic blood pressure is measured after 5 minutes of rest in seated position by Collon Press mate (BP-8800, Komaki-City). HbA1c will be measured by High Performance Liquid Chromatography and total cholesterol and HDL-cholesterol by means of enzymatic techniques (Boehringer-Mannheim, Mannheim, Germany). Age, duration of diabetes, sex, ethnicity, level of education, marital or cohabiting status, employment status, family history of T2DM and CVD and smoking status are included in the self-reported questionnaires. Smoking status will be distinguished in non-smoker, ex-smoker or current smoker including number of cigarettes per day.

### Sample size

The primary outcome measure is risk perception. Because we do not have the availability of a validated questionnaire it is not possible to use data of previous studies for the purpose of the power calculation. Therefore, we choose to use Cohen's effect sizes [50]. The power calculation is based on a difference of 0.5 standard deviations between the intervention and control group. The calculation is based on the Student's t-test for two independent groups.

The result is that we need 84 patients in each group to detect a difference of half a standard deviation with a power of 90%. Taking into account a possible dropout rate of 30% the number of patients in each group must be 120. In 2008, approximately 40 newly diagnosed patients per month were registered at the DCS, which means 480 patients per year. It is expected that not all patients are eligible and willing to participate in the study and therefore we preserve an inclusion period of 12 months.

### Analyses

Comparability between the two groups will be assessed at baseline. On the basis of an intention-to-treat analysis, differences in changes between the intervention and the control group are measured with 95% confidence intervals at 2 and 12 weeks for both primary and secondary outcomes. For dichotomous outcome variables multilevel logistic regression analyses will be used. To calculate differences between continuous variables linear regression analyses will be used. If there are any relevant differences between the groups, we will adjust data for these factors (i.e. age, gender, diabetes duration, level of education and family history regarding T2DM and CVD).

## Discussion

This article contains an extensive description of the design of the @RISK Study: an intervention to investigate the effects of a newly designed risk communication protocol on risk perception, attitude and intention to change lifestyle behaviour. We expect that due to the risk communication intervention, patients' appropriateness of risk perception will improve in a way that they will understand the meaning of their absolute 10 year risk to develop CVD, and subsequently develop a more positive attitude and intention towards lifestyle behaviour change. Risk communication is very relevant and important for patients with diabetes because nowadays patients are expected to make their own choices and decisions concerning their diabetes care.

Only a few studies on the effectiveness of risk communication on changing patients' risk perception and attitude on lifestyle changes have been performed. Brenner et al. performed a randomised controlled trial on risk communication in patients with hypertension and a CVD risk > 10%. The intervention included educating patients of their 10-year risk of myocardial infarction or death, illustrated by a graphical representation of the risk, and three follow-up phone calls by a physician or study nurse. This resulted in behaviour modification and in a significant reduction in CVD risk [51,52]. A randomised controlled trial by Koelewijn-van Loon et al. found that appropriateness of risk perception improved and anxiety reduced. They found no improvement in lifestyle in patients with high risk of developing CVD. As the risk communication was part of a large intervention study, it is unclear if the risk communication itself has caused the improvement [46,53].

To our knowledge, evidence on this topic for patients with T2DM is scarce and this study might have an important contribution to the research in this field. Only two studies have been performed on patients with T2DM. Asimakopoulou et al. studied the impact of risk communication by means of a combination of visual tools of developing CHD in patients with T2DM. They found that patients improved their risk perception and they were able to recall their risk after six weeks [54]. Edwards et al. evaluated different risk presentation formats in patients with diabetes and showed that patients found graphical representations helpful and had no clear preference for which graphical tool, however it did not change patients' ability to make decisions on their health [18]. These two studies were not based on theory, which makes it difficult to untangle the mechanism of risk perception in relation to self-management. In addition, the purpose of the study of Edwards et al. was to investigate which kind of graphical information was most helpful and not really the risk communication itself.

The key element of this trial is the use of a theoretical framework, based on the Theory of Planned Behaviour [38,39] and the Self-regulation Theory [35-37]. Elements of the intervention are based on the theoretical framework. Communication of absolute risk is based on the illness perception 'cause' of the Self-regulation Theory and the communication by means of a positive message is focused on the 'controllability' of the theory. This message tells patients that they can control their disease themselves.

The population diagram used in this study was already found to be feasible in the IMPALA study [46], which is a trial on the implementation of a nurse-led intervention for cardiovascular risk management in primary care. Their risk communication tool was tested in a pilot study. The risk tool in IMPALA also consisted of a bar chart to explain the possibilities for risk reduction also by relative risk reduction, but process evaluation showed that this was hardly used by the nurses in the IMPALA study.

In this study, much attention is paid to the diabetes nurses' and dieticians' skills to perform the intervention by means of a training program and pilot study. The main strength of this part of the study is the availability of an experienced communication coach who has been performing 'coaching-on-the-job'. This will improve the quality and standardisation of the intervention, rather than supervision in groups, lacking personal feedback to the caregivers.

A limitation of the study design is that we were not able to divide the diabetes nurses and dieticians into two groups: one group that performs the intervention and one group that takes care of the control group. The reason for this limitation is a practical one: we implemented the study into the DCS in which diabetes nurses and dieticians work part-time. If we trained only half of the diabetes nurses and dieticians employed at the DCS, the intervention could not be performed every day of the week. This would cause problems in scheduling the patients, which is likely to influence their willing to participate in the study. Therefore, we decided to train all diabetes nurses and dieticians. To increase treatment fidelity we developed both an intervention and a control group protocol. In addition, we will make tape recordings to assess treatment fidelity.

This study started at the end of 2008 with the training of the diabetes nurses and dieticians, followed by the pilot study. The inclusion of patients started in March 2009 and will continue through February 2010. Results will become available at the end of 2010.

## Abbreviations

CVD: cardiovascular disease; DCS: Diabetes Care System West-Friesland; GP: general practitioner; T2DM: type 2 diabetes mellitus; UKPDS: United Kingdom Prospective Diabetes Study.

## Competing interests

The authors declare that they have no competing interests.

## Authors' contributions

LW is responsible for the data-collection and wrote the manuscript. All authors contributed to the development of the design of the study and have read and approved the final manuscript.

## Pre-publication history

The pre-publication history for this paper can be accessed here:

http://www.biomedcentral.com/1471-2458/10/457/prepub
